# Association of genetic variants in migraineurs with and without restless legs syndrome

**DOI:** 10.1002/acn3.51186

**Published:** 2020-09-12

**Authors:** Guan‐Yu Lin, Yu‐Kai Lin, Chih‐Sung Liang, Jiunn‐Tay Lee, Chia‐Lin Tsai, Kuo‐Sheng Hung, Wen‐Jie Luo, Chia‐Kuang Tsai, Yu‐Wei Hsu, Tsung‐Han Ho, Fu‐Chi Yang

**Affiliations:** ^1^ Department of Neurology Tri‐Service General Hospital National Defense Medical Center Taipei Taiwan; ^2^ Department of Neurology Songshan Branch Tri‐Service General Hospital National Defense Medical Center Taipei Taiwan; ^3^ Department of Psychiatry Beitou Branch Tri‐Service General Hospital National Defense Medical Center Taipei Taiwan; ^4^ Center for Precision Medicine and Genomics Tri‐Service General Hospital National Defense Medical Center Taipei Taiwan

## Abstract

**Objective:**

Several single‐nucleotide polymorphisms (SNPs) are associated with restless legs syndrome (RLS). This study investigated whether or not additional SNP variants increase the risk of RLS in migraineurs and in migraine with aura (MA) and migraine without aura (MoA) subgroups.

**Methods:**

Migraineurs with and without RLS were genotyped using an Affymetrix array. We performed association analyses for the entire cohort and the MA and MoA subgroups, which were divided further into episodic migraine (EM) and chronic migraine (CM). Potential correlations between SNPs and clinical indices in migraineurs with RLS were examined by multivariate regression analysis.

**Results:**

The rs77234324 and rs79004933 SNPs were found in migraineurs with (*P* = 2.57E‐07) and without (*P* = 3.03E‐07) RLS. The A allele frequency for rs77234324 (on *LGR6*) was 0.1321 in migraineurs with RLS and 0.0166 in those without RLS (odds ratio, 8.978). The T allele frequency for rs79004933 (in the intergenic region) was 0.1981 in migraineurs with RLS and 0.0446 in those without (odds ratio, 5.281). rs2858654, rs76770509, rs4243475 in *UTRN,* rs150762626, and rs2668375 were identified in migraine with and without RLS in the MoA subgroup (*P* = 7.56E‐09, *P* = 2.30E‐08, *P* = 1.19E‐07, *P* = 6.86E‐07, and *P* = 8.05E‐07, respectively). There was a suggestion of an association between rs10510331 (*P* = 1.50E‐06) and CM and EM in patients with MoA and RLS. Multivariate regression showed a significant relationship between rs79004933 and the Beck Depression Inventory score.

**Interpretation:**

rs77234324 in *LGR6* and rs79004933 in the intergenic region were associated with RLS in migraineurs. Five SNPs increased the risk of RLS in patients with MoA.

## Introduction

Restless legs syndrome (RLS) is a sleep‐related sensorimotor disorder with a female predominance and is characterized by unpleasant leg sensations and an irresistible urge to move. Symptoms of RLS typically occur during rest and at night‐time and resolve with leg movement.^1^ The reported prevalence of RLS ranges from 4% to 29% and is higher in Western countries (5%–10%) than in Asian populations (1%–4%).^2^

Migraine is a prevalent primary headache, affecting approximately 10%–20% of the world's population with a female‐to‐male predominance of 2–3:1. It is typically characterized by recurrent attacks of moderate‐to‐severe throbbing or pulsating pain on one side of the head that last for 4–72 h and may be associated with photophobia, phonophobia, and/or nausea and vomiting, which is aggravated by physical activity.^3^, ^4^ Accumulating evidence shows that certain diseases, including cardiovascular disease, psychiatric disorders, epilepsy, and pain disorders, are associated with migraine.^5^ A systematic review found that RLS was more common in migraineurs (17.0%) than in nonmigraineurs (7.0%).^6^ Suzuki et al. reported that migraineurs with RLS had higher Migraine Disability Assessment (MIDAS) scores and poorer sleep quality with more symptoms of depression than their counterparts without RLS.^7^ Migraine and RLS may share some common pathophysiological mechanisms involving dopaminergic dysfunction and disturbed iron metabolism and have known genetic risk factors. Although Lucchesi et al. found that RLS was more common in individuals with chronic migraine (CM) than in those with episodic migraine (EM), no conclusions can be drawn based on the limited data currently available.^6^, ^8^ Similarly, Bilgehan et al. found that migraine, particularly migraine with aura (MA), was more common in patients with RLS^9^; however, d’Onofrio et al. did not.^10^ Therefore, the relationship between MA/migraine without aura (MoA) and RLS has not been confirmed.

RLS has a strong genetic basis with a heritability of approximately 50%. Genome‐wide association studies (GWAS) in European populations found six RLS susceptibility loci, including the Meis homeobox 1 gene (*MEIS1*), protein tyrosine phosphatase receptor type D gene (*PTPRD*), BTB domain containing 9 gene (*BTBD9*), TOX high mobility group box family member 3 gene (*TOX3*), mitogen‐activated protein kinase 5/SKI family transcriptional corepressor 1 gene (*MAP2K5*/*SKOR1*), and an intergenic region on chromosome 2p14, the strongest candidates being variants of *MEIS1*, *PTPRD*, and *BTBD9*.^11^ One study demonstrated a significant association of *MEIS1* with RLS and that the single‐nucleotide polymorphism (SNP) rs2300478 increased the risk of RLS by 1.42‐fold in a migraine cohort.^2^ Schormair et al. confirmed an association of RLS with rs4626664 in *PTPRD* in Caucasians^12^; Lin et al. also described an association between the *PTPRD* variant rs4626664 and uremic RLS.^13^ However, Kim et al. found that rs3923809 and rs9296249 in *BTBD9,* but not rs4626664 in *PTPRD*, were implicated in the pathogenesis of RLS in Koreans.^14^

**Figure 1:**
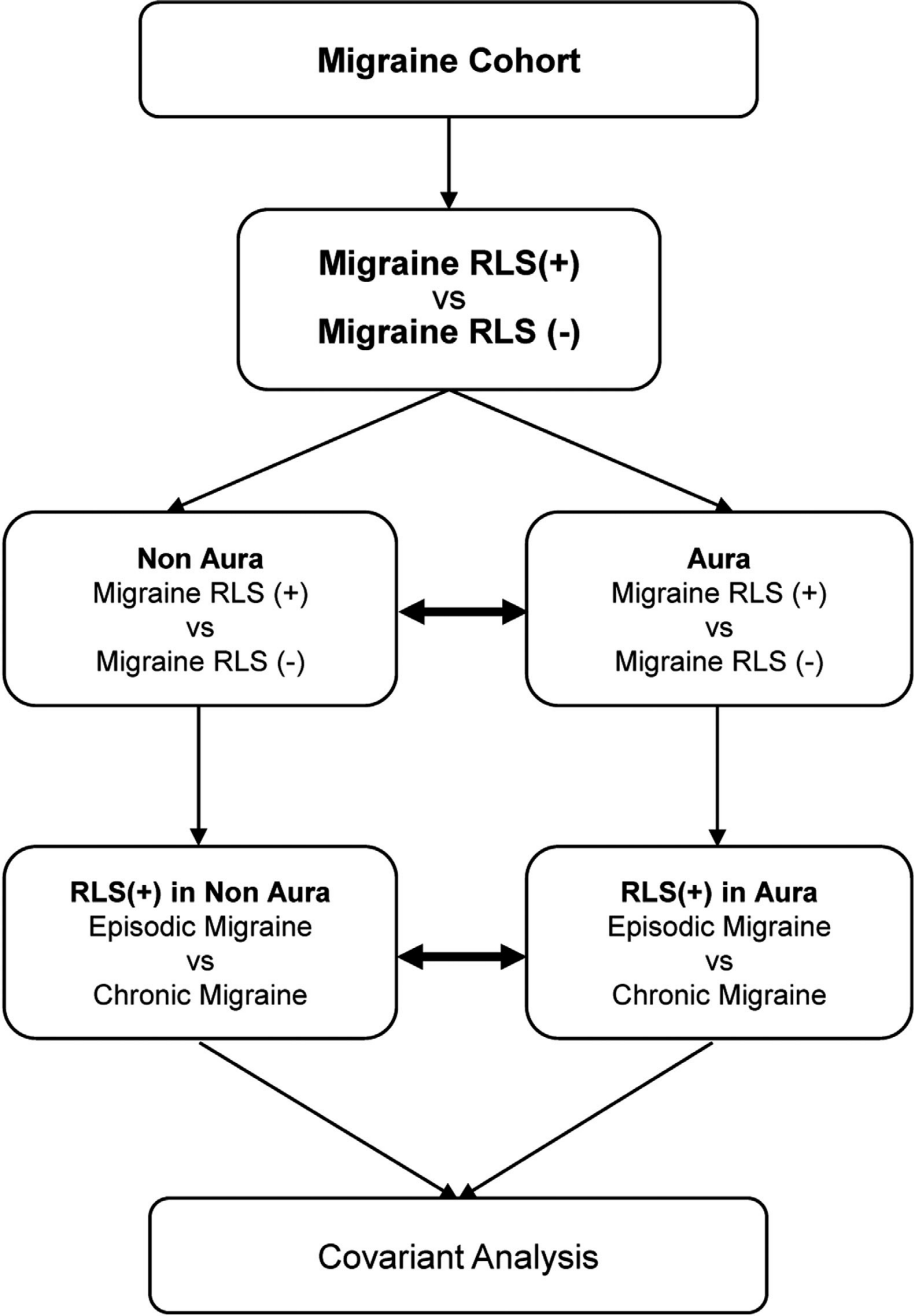
Flowchart showing the process used to compare the study data. We collected the genotypes and phenotypes of 233 patients with migraine and divided them into an RLS+ (experimental) group and an RLS‐ (control) group for comparison. The results are shown in Table 2. We selected patients with aura from the migraine cohort and divided them into an RLS+ (experimental) group and an RLS‐ (control) group for comparison. Patients without aura from the migraine cohort were also divided into an RLS+ (experimental) group and an RLS‐ (control) group for comparison. The two groups of patients with and without aura were similarly divided into a chronic migraine group and a episodic migraine group for comparison. The results are shown in Table 3. Multivariate regression analyses were performed for the Pittsburgh Sleep Quality Index, Beck Depression Inventory, and the Hospital Anxiety and Depression Scale scores. RLS, restless legs syndrome

This study investigated whether or not there are additional causative gene loci that are associated with comorbid RLS and migraine. SNPs previously identified in GWAS to be associated with RLS were examined in Taiwanese migraineurs according to subtype (MA vs. MoA and CM vs. EM) (Figure 1).

## Materials and Methods

### Patients

The study was performed in a cohort of 233 patients recruited from the neurology outpatient department at Tri‐Service General Hospital (TSGH) between October 2018 and June 2019. The study protocol was approved by the TSGH Institutional Review Board. All patients provided written informed consent before enrollment.

Each study participant completed a screening questionnaire and was subsequently interviewed by a board‐certified neurologist and headache specialist (FCY), who made a diagnosis based on the criteria in the third edition of the International Classification of Headache Disorders.^4^ The study sample were then divided into a group with CM (≥15 episodes per month; *n* = 71) or EM (<15 episodes per month; *n* = 162). Sixty (26%) of the 233 study participants had MA and 169 (73%) had MoA.

### Patient assessment

#### Evaluation of migraine

All patients completed a standardized demographic questionnaire and the MIDAS,^15^ Pittsburgh Sleep Quality Index (PSQI),^16^ Beck Depression Inventory (BDI),^17^ and Hospital Anxiety and Depression Scale (HADS)^18^ questionnaires.

The MIDAS is a 5‐item questionnaire that evaluates headache‐related disabilities over the previous 3 months and has a score range of 0–270.^15^ The PSQI includes 19 self‐rated items combined into seven components and is designed to assess sleep quality during the previous month; it has a score range of 0–21, with a total PSQI score ≥ 6 indicating sleep disturbance.^16^ The BDI scores range from 0 to 63, and individuals who score ≥ 18 are classified as depressed.^17^ The HADS has seven items related to anxiety and depression and has maximum individual subscale scores of 21.^18^

### Evaluation of RLS

RLS was diagnosed according to the following criteria proposed by the International RLS Study Group: (1) an urge to move the legs, usually accompanied by unpleasant sensations in the legs; (2) an urge to move or uncomfortable sensations that begin or worsen during rest, including lying down or sitting; (3) an urge to move or uncomfortable sensations that are partially or totally relieved by leg movements; (4) compared with daytime, an urge to move or uncomfortable sensations that worsen in the early evening or at night; and (5) no other medical or behavioral conditions (e.g., venous stasis, leg edema, myalgia, arthritis, positional discomfort, habitual foot tapping, or leg cramps) that could explain the above features.^1^

Patients with a history of physical, cognitive, degenerative neurological disease, or severe head injury were excluded. All participants were required to receive neurological, electromyographic, and Doppler ultrasound examinations. Patients with common causes of secondary RLS, including anemia, a serum ferritin level < 50 ng/ml, and a serum creatinine > 1.5 mg/dl, were also excluded, as were those who were pregnant. Only patients with idiopathic RLS were enrolled in the study.

### Genotyping and quality control

In collaboration with the National Centre for Genomics Medicine, Academia Sinica, Taiwan, genomic deoxyribonucleic acid (DNA) for the GWAS was extracted from peripheral blood obtained from our patients for genotyping with the Affymetrix Axiom Genome‐Wide TWB 2.0 array, which contains 446,000 SNPs that are sufficient to represent the Taiwanese genotypic background, about 105,000 SNPs that are clinically significant, and further disease‐related SNPs added by Thermo Fisher Scientific over the years. In total, approximately 710,525 SNPs are related to drug response, metabolism, and detection of gene copy number variations. In August 2019, Academia Sinica’s Taiwan Precision Medicine Initiative reached the milestone of 100,000 samples genotyped using this chip.^19^, ^20^ Blood was collected in 5‐ml EDTA vacutainers (BD, Plymouth, UK). Genomic DNA was extracted using the QIAamp DSP DNA Mini Kit in the QIAsymphony platform (Qiagen, Hilden, Germany). After extraction, the quality of the DNA was assessed using a NanoDrop One spectrophotometer (Thermo Fisher Scientific, Waltham, MA, USA). Raw Axiom TWB 2.0 SNP array signal CEL files were transformed to genotyping data (tped and tfam) using Analysis Power Tools. The quality control procedures were conducted using PLINK.^21^ The quality of genotyping was evaluated by a genotype calling rate of <97% and Hardy‐Weinberg equilibrium (*P* < 0.00001). SNPs that failed to pass the quality control (calling rate < 97% or Hardy‐Weinberg equilibrium < 0.00001) were excluded.

### Statistical analysis

Demographic data for the migraine groups and subgroups were compared using Fisher’s exact test or the *t*‐test according to the type of variable. SNP associations were examined with PLINK and included the following groups: (1) migraine with RLS versus migraine without RLS, (2) migraine with RLS versus migraine without RLS in patients with MoA, (3) migraine with RLS versus migraine without RLS in patients with MA with separation of (4) EM versus CM in patients with MoA from the migraine with RLS group and (5) EM versus CM in patients with MA from the migraine with RLS group. We were also interested in the correlation between SNPs and the index for clinical identification of poor sleep quality, depression, and anxiety. Significant variants were selected from the analysis of results for patients who had migraine with or without RLS for additional analyses of several indices, including the PSQI, BDI score, and HADS scores by multivariate regression.

## Results

### Demographics

Table 1 shows the demographic characteristics of the study participants according to whether or not they had RLS. There were no significant differences in sex, age, body mass index, education level, frequency of migraine, EM/CM, or MIDAS scores. However, patients with MA had significantly higher BDI, PSQI, HADS‐anxiety, and HADS‐depression scores if they had comorbid RLS (*P* < 0.05).

**Table 1:** Demographic and clinical data

	Migraine with RLS	Migraine without RLS	*P*‐value
Migraine cohort	*n* = 53	*n* = 180	NA
With aura/without aura	26/27	34/142*	<0.001
EM/CM	33/20	129/51	0.23
Migraine frequency	10.63 ± 7.31	8.78 ± 6.12	0.13
Sex (male/female)	11/41*	27/113*	0.84
Age, years	43.78 ± 11.01	40.06 ± 11.37	0.058
Body mass index	22.61 ± 3.40	23.64 ± 4.50	0.15
Education, years	13.49 ± 3.41	14.11 ± 3.17	0.42
IRLSSG score	13.28 ± 6.65	NA	NA
MIDAS score	23.40 ± 19.18	23.69 ± 16.22	0.92
PSQI score	11.87 ± 4.11	9.35 ± 3.96	<0.001
BDI score	15.66 ± 8.70	8.57 ± 8.55	<0.001
HADS anxiety score	7.60 ± 3.95	6.11 ± 4.55	<0.001
HADS depression score	11.9 ± 4.38	9.41 ± 4.16	0.039

Fisher exact test and the *t*‐test were performed as appropriate. BDI, Beck Depression Inventory; CI, confidence interval; CM, chronic migraine; EM, episodic migraine; HADS: Hospital Anxiety and Depression Scale; IRLSSG, International RLS Study Group; MIDAS, Migraine Disability Assessment Scale; OR, odds ratio; PSQI, Pittsburgh Sleep Quality Index.

* The sum of these value does not match the total number because of missing data.

### Association of RLS variants in patients with migraine

Fifty‐three of the 233 study participants had comorbid RLS and 180 did not. Association analysis found that two SNPs, rs77234324 and rs79004933, had a *P*‐value < 1E‐7 (*P* = 2.57E‐07 and *P* = 3.03E‐07, respectively) for migraine with and without RLS (Table 2). The A allele frequency for rs77234324 in *LGR6* was 0.1321 in migraineurs with RLS and 0.0166 in those without RLS (odds ratio 8.978). The T allele frequency for rs79004933 in the intergenic region was 0.1981 in patients with comorbid migraine and RLS and 0.0446 in patients with migraine only (odds ratio 5.281).

**Table 2:** Association between migraine with RLS and migraine without RLS

SNP	Position (GRCh38.p12)	Gene	Risk allele	Risk allele frequency		*P*‐value	OR
				Migraine with RLS (*n* = 53)	Migraine without RLS (*n* = 180)		
rs77234324	Chr1:202286197	LGR6	A	0.1321	0.01667	2.57E‐07	8.978
rs79004933	Chr3:31257437	intergenic	T	0.1981	0.04469	3.03E‐07	5.281

Patients in the migraine with RLS and migraine without RLS groups were compared using Plink. Significant variations with an empirical *P*‐value < 1E‐7 are listed with the allele frequency and odds ratio. RLS, restless legs syndrome; SNP, single‐nucleotide polymorphism.

### Association of RLS variants in the MA and MoA subgroups

The migraine cohort was divided into MA and MoA subgroups; association analysis identified five SNPs (rs2858654, rs76770509, rs4243475, rs150762626, and rs2668375) between MoA with RLS and MoA without RLS (*P* = 7.56E‐09, *P* = 2.30E‐08, *P* = 1.19E‐07, *P* = 6.86E‐07, and *P* = 8.05E‐07, respectively). The genotype distributions of four SNPs were in the intergenic region and rs4243475 was in *UTRN* (Table 3). In contrast, no variants reached the threshold *P*‐value between MA with RLS and MA without RLS.

**Table 3:** Association between migraine and RLS in the subgroup with migraine without aura

Group	SNP	Position (GRCh38.p12)	Gene	Risk allele	Risk allele frequency		*P*‐value	OR
					Migraine with RLS	Migraine without RLS		
					(*n* = 27)	(*n* = 142)		
MoA	rs2858654	Chr22:49267114	Intergenic	T	0.2222	0.02465	7.56E‐09	11.31
	rs76770509	Chr12:102208544	Intergenic	G	0.1296	0.003521	2.30E‐08	42.15
	rs4243475	Chr6:144793799	UTRN	A	0.3269	0.07394	1.19E‐07	6.083
	rs150762626	Chr11:56306080	Intergenic	C	0.2593	0.05282	6.86E‐07	6.277
	rs2668375	Chr12:17526379	Intergenic	A	0.5185	0.2007	8.05E‐07	4.289

MoA, migraine without aura; OR, odds ratio; RLS, restless legs syndrome

### Comparison of RLS‐associated variants between CM and EM in the MA and MoA subgroups

We also compared EM and CM in patients with MoA and RLS and in those with MA and RLS; the SNP rs10510331 (LOC105376942, chr3: 6171812) showed a suggestive *P*‐value of 1.50E‐06 (odds ratio 57.4). The G allele frequency was 0.5833 for CM and 0.02381 for EM, in CM versus EM in none of the aura association test, and none in CM versus EM in aura association test (not shown).

### Associations between an RLS variant and clinical variables

Multivariate regression analyses of the PSQI, BDI, HADS‐anxiety, and HADS‐depression scores identified a significant association between rs79004933 (chr3: 31257437) and the BDI score (*P* = 0.03821, odds ratio 1.226, 95% confidence interval 1.011–1.486; not shown).

### Replication study

Several loci have previously been identified to be associated with an increased risk of RLS.^2^ Of 13 SNPs in six RLS‐associated loci (*MEIS1*, *PTPRD*, *BTBD9*, *TOX3*, *MAP2K5*, and an intergenic region on chromosome 2p14), 10 were not detected on the TWB 2.0 array and two of three SNPs, rs2300478 (*MEIS1*, chr2: 66554321) and rs4626664 (*PTPRD*, chr9: 9261737) were detected more often in migraineurs with RLS than in their counterparts without RLS (*P* = 0.01142 and *P* = 0.02097, respectively). Furthermore, rs4626664 (*PTPRD*, chr9: 9261737) was detected more often in MoA with RLS than in MoA without RLS (*P* = 0.02442; Table 4).

**Table 4:** Replication of findings in previous studies.^2^

SNP	Gene	Risk allele	Group	Migraine with RLS	Migraine without RLS	*P*‐value
rs2300478	MEIS1	G	Migraine cohort	0.3302	0.2111	0.01142
			Aura ‐	0.3148	0.2183	0.1245
rs6747972	Intergenic	A	Migraine cohort	0.3491	0.4056	0.2951
			Aura ‐	0.3333	0.4085	0.301
rs4626664	PTPRD	A	Migraine cohort	0.5377	0.4111	0.02097
			Aura ‐	0.5741	0.4085	0.02442

Variations were found by comparing within migraineurs with RLS versus those without RLS and between MoA with and without RLS.

## Discussion

In this study, we found that migraineurs with SNPs rs77234324 (in *LGR6*) and rs79004933 (an intergenic variant on chromosome 3) were more susceptible to RLS. We also identified five RLS‐associated SNPs (rs2858654, rs76770509, rs150762626, rs2668375, and rs4243475 in *UTRN*, which encodes utrophin) in individuals with migraine, especially MoA. The SNP rs79004933 associated with RLS in migraineurs was also associated with higher BDI total scores. In the replication portion of the study, we found that two SNPs (rs2300478 in *MEIS1* and rs4626664 in *PTPRD*) increased the risk of RLS in the migraine cohort but only rs4626664 in *PTPRD* increased the risk of RLS in individuals with MoA.

An updated systematic review and meta‐analysis demonstrated that patients with comorbid migraine had more severe symptoms of RLS.^6^ Although the exact mechanisms of the association between migraine and RLS are not clearly understood, several lines of evidence suggest that RLS and migraine might share some clinical features and an underlying pathophysiology. Disturbance of iron metabolism and dopaminergic dysfunction have been identified in both conditions. Deposition of iron in the brain has been reported in migraineurs, and repeated attacks have been associated with increased accumulation of iron. In contrast, iron deficiency has been implicated in the pathogenesis of RLS. Moreover, dopaminergic (premonitory) symptoms are more common in patients with migraine and RLS than in those with migraine alone. Dysfunction of A11 dopaminergic neurons in the hypothalamospinal tract is also strongly implicated in the pathophysiology of RLS.^22^

In recent years, a variety of genetic analyses, mostly GWAS, have been performed to clarify the link between RLS and migraine. A previous study identified 13 SNP markers at six RLS‐related loci (*MEIS1*, *BTBD9*, *MAP2K5*, *PTPRD*, *TOX3*, and an intergenic region on chromosome 2p14) in Caucasian populations, and the replication portion of the study indicated an association between rs2300478 in *MEIS1* with a significantly increased risk of RLS in an Asian migraine cohort.^2^ In our study, we found that two SNPs (rs77234324 in *LGR6* and rs79004933, an intergenic variant in chromosome 3) increased the susceptibility of migraineurs to RLS. *LGR6*, a member of the rhodopsin‐like seven transmembrane domain receptor superfamily, potentially functions as a tumor suppressor gene and was found to be mutated when its promoter region was hypermethylated in around 20%–50% of patients with colon cancer and has been correlated with refractory epilepsy.^23^, ^24^ Further research is needed to determine the pathomechanism of the RLS‐related variant in *LGR6* in individuals with migraine.

Cho et al. performed GWAS for RLS in a Korean population and found that an rs9390170 polymorphism in *UTRN* was a genetic marker of susceptibility to RLS.^25^ The human gene for utrophin is located on chromosome 6 and encodes a 395‐kDa protein that is highly homologous with dystrophin.^26^ Utrophin was found to be expressed in almost all tissues and mainly localized in the synaptic regions of skeletal muscle fibers.^27^ Duchenne’s muscular dystrophy is a condition in which a lack of dystrophin results in progressive loss of muscle strength and ultimately to death from respiratory or cardiac failure.^28^ Upregulated expression of utrophin has been found in dystrophinopathies and other disorders of muscle, including congenital myopathies, inflammatory myopathies, diabetic neuropathies, amyotrophic lateral sclerosis, spinal muscular atrophies, and minimal change myopathies.^29^ Moreover, utrophin might play a role in the dystrophin glycoprotein complex, which comprises an array of glycoproteins at the neuromuscular junction.^30^ The dystrophin glycoprotein complex is also distributed in the brain, blood–brain barrier, and choroid plexus and is associated with neuronal nitric oxide synthase (nNOS).^31^ Nitric oxide might be involved in the pathophysiological link between RLS and utrophin because of the close relationship between NOS and utrophin.^32^ West et al. reported that nNOS modulated dopaminergic neurotransmission in the striatum,^33^ whereas Patton et al. suggested that activation of the hypoxia pathway in individuals with RLS increases production of nNOS and is also closely related to iron metabolism, dysregulation of which is involved in RLS.^34^ Therefore, we speculated that dysfunction of utrophin was involved in other neuromuscular diseases, including RLS. To our knowledge, this study is the first to demonstrate that the rs4243475 in *UTRN* increases the risk of RLS in patients with MoA.

Previous researchers have identified RLS‐associated SNP variants in several genes, including *PTPRD*, *BTBD9*, *MEIS1*, *MAP2K5*, and *TOX3*, and in an intergenic region on chromosome 2p14.^2^, ^11^ The mechanism of the link between these genes and a higher RLS risk might be related to aberrant iron homeostasis and an imbalance in the dopaminergic system. A shared pathogenesis between RLS and migraine might explain why these SNP variants of RLS are found in migraineurs.^35^ In the replication part of our study, three SNPs at RLS risk loci (*MEIS1*, *PTPRD*, and an intergenic region on chromosome 2p14) were genotyped in 233 patients with migraine and in 53 with comorbid RLS. Like Fuh et al,^2^ we found that presence of the *MEIS1* variant rs2300478 was associated with an increased risk of RLS in our migraine cohort but not specifically in the subgroup with MoA. A significant association of rs4626664 in *PTPRD* with RLS in Caucasians was reported by Schormair et al.^12^ and with uremic RLS by Lin et al.^13^ However, no significant association of the RLS‐related variant in the intergenic region on chromosome 2p14 was found in the migraine cohort or in the MoA subgroup. In our study, we determined that rs2300478 in *MEIS1* and rs4626664 in *PTPRD* increased the risk of RLS in the entire cohort; furthermore, the SNP rs4626664 in *PTPRD* was significantly associated with RLS in patients with MoA.

Bilgehan et al.^9^ indicated that migraine and MA were significantly more common in patients with RLS; as in our previous study, we observed an association between the prevalence of RLS and that of migraine, especially MA.^21^ We also showed that migraineurs with RLS had poor sleep quality more often than those without RLS, which corroborated our previous finding that RLS independently predicted sleep disturbance (PSQI score ≥ 6).^36^ Furthermore, individuals with migraine and RLS reportedly have more severe symptoms of anxiety and depression and higher BDI total scores than those without RLS.^6^, ^7^, ^22^ The findings of this study suggest that individuals with migraine and RLS are more likely to have sleep disturbance and emotional disorders than those without RLS. Multivariate regression analysis showed that one SNP (rs79004933) was correlated with the BDI score in patients with migraine and RLS. When considering the heavy burden of migraine and RLS in the general population, it is important to diagnose and treat psychiatric and sleep problems in patients with migraine, especially those with RLS.

The strengths of this study are the well‐controlled design of the genetic analysis and robust statistical analysis. Moreover, the diagnoses of migraine and RLS were made by qualified physicians according to a strictly audited protocol using the third edition of the International Classification of Headache Disorders^4^ for migraine and the International RLS Study Group recommendations for RLS.^1^ Affymetrix's Axiom Genome‐Wide TWB 2.0 array covers a highly representative sample of the gene pool in Taiwan. However, there were also several limitations. First, all study participants were recruited from the neurology outpatient department of a tertiary hospital, which might limit the generalizability of our findings. Second, depression was self‐reported based on a questionnaire rather than a psychiatric interview. Nevertheless, our data for the relationship between RLS, migraine, and depression were in accordance with a recent literature review.^6^ Finally, the cohort sample was relatively modest. However, this drawback could be offset by the precise diagnostic methods used. Further studies in larger samples are needed to replicate and extend our present knowledge of genetic risk factors in Asian populations.

In conclusion, this study found that rs77234324 (in *LGR6*) and rs79004933 (an intergenic variant in chromosome 3) increased the likelihood of RLS in a cohort of patients with migraine. Furthermore, rs79004933 was correlated with the BDI score in migraineurs with comorbid RLS. Five SNPs (rs2858654, rs76770509, rs4243475 in *UTRN,* rs150762626, and rs2668375) were associated with RLS in the subgroup with MoA. Further studies are warranted to investigate whether or not additional genes may be involved in the shared pathogenesis of RLS and migraine.

## Conflict of Interest

The authors declare no conflicts of interest.

## Author Contributions

F‐CY had full access to all the data in the study and took responsibility for its integrity. G‐YL, Y‐KL, C‐SL, and F‐CY contributed to the study concept and design, acquisition, and interpretation of data. C‐LT, J‐TL, K‐SH, and C‐KT contributed to the analysis of the data. G‐YL and F‐CY participated in drafting the manuscript. All authors contributed to the collection and execution of the study. All authors have read and approve of the final version of the manuscript.
